# The Molecular Mechanism of Natural Killer Cells Function and Its Importance in Cancer Immunotherapy

**DOI:** 10.3389/fimmu.2017.01124

**Published:** 2017-09-13

**Authors:** Sourav Paul, Girdhari Lal

**Affiliations:** ^1^National Centre for Cell Science, Pune, India

**Keywords:** natural killer cells, tumor microenvironment, inflammation, innate immune cells, cancer immunotherapy

## Abstract

Natural killer (NK) cells are innate immune cells that show strong cytolytic function against physiologically stressed cells such as tumor cells and virus-infected cells. NK cells show a broad array of tissue distribution and phenotypic variability. NK cells express several activating and inhibitory receptors that recognize the altered expression of proteins on target cells and control the cytolytic function. NK cells have been used in several clinical trials to control tumor growth. However, the results are encouraging only in hematological malignancies but not very promising in solid tumors. Increasing evidence suggests that tumor microenvironment regulate the phenotype and function of NK cells. In this review, we discussed the NK cell phenotypes and its effector function and impact of the tumor microenvironment on effector and cytolytic function of NK cells. We also summarized various NK cell-based immunotherapeutic strategies used in the past and the possibilities to improve the function of NK cell for the better clinical outcome.

## Introduction

Natural killer (NK) cells are a group of innate immune cells that show spontaneous cytolytic activity against cells under stress such as tumor cells and virus-infected cells. After activation, NK cells also secrete several cytokines such as interferon-γ (IFN-γ), tumor necrosis factor-α (TNF-α), granulocyte macrophage colony-stimulating factor (GM-CSF), and chemokines (CCL1, CCL2, CCL3, CCL4, CCL5, and CXCL8) that can modulate the function of other innate and adaptive immune cells. NK cells are identified as CD3^−^NK1.1^+^ cells in C57BL/6, FVB/N, and NZB strains of mice. BALB/c, CBA/J, AKR, C3H, DBA/1, DBA/2, NOD, SJL, and 129 strains of mice do not express NK1.1 and NK cells in these mice can be identified as CD3^−^CD49b^+^ cells. NK cells in human are identified as CD3^−^CD56^+^ cells. They represent 2–7% of lymphocytes in mouse peripheral blood (PB) and 5–15% of human peripheral blood mononuclear cells (PBMCs). NK cells are present in the skin, gut, liver, lung, uterus, kidney, joints, and breast under physiological conditions. NK cells constitute about 20–30% of total hepatic lymphocytes and 10% of lymphocytes in healthy human liver and lung, respectively (1). The specific subset of NK cell is reported to control the development at the fetal-maternal interface during the first trimester of the pregnancy, and it constitutes about 50–90% of total lymphoid cells in the uterus (2, 3). These uterine NK cells secrete IL-8, vascular endothelial growth factor (VEGF), stromal cell-derived factor-1, and interferon gamma-inducible protein-10 (IP-10) which help in tissue building, remodeling, and angiogenesis (4). NK cells in human placenta do not show killer activity but assist in establishing immunosuppression and tolerance to fetus allograft. Similar to T and B cells, NK cells also develop from common lymphoid progenitor cells (5). Although bone marrow is the primary site of NK cell development (6), they can also develop in the liver and thymus (7). The development of NK cells progresses through various stages of maturation, expansion, and acquisition of specific receptors. All NK receptors are germ-line encoded and independent of RAG-mediated recombination (8). Multiple factors such as cell-intrinsic signals (transcription factors) and external signals (cytokines and growth factors) govern the development of NK cells. NK cells constitute the major component of an innate immune system and play the crucial role in shaping the early immune response to viral infection and tumors and also in organ transplantation (9). In this review, we discussed what are inhibitory and activating molecules present on NK cells and how they control NK cell function, how do NK cell function in the tumor microenvironment, use of NK cell as adoptive cellular therapy to control cancer and what are strategies to improve NK cell antitumor function.

## Effector and Regulatory Phenotype of NK Cells

**Figure 1:**
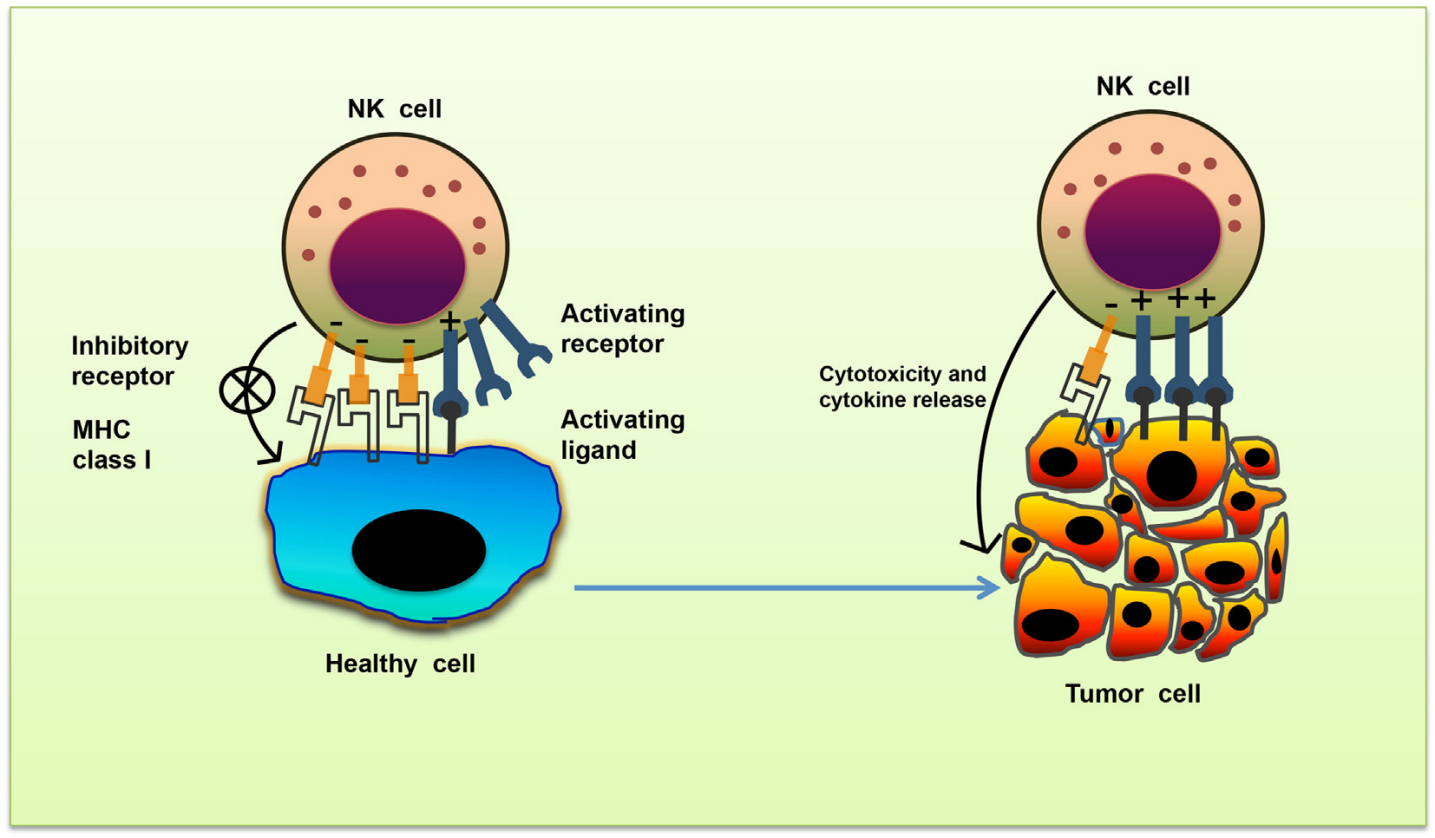
Missing-self recognition of target cells. The activating and inhibitory receptor signaling regulates the natural killer (NK) cells activation. Cells undergoing stress such as tumor cells lose their MHC class I molecules, a ligand for inhibitory receptors on NK cells. At the same time, they acquire stress-associated molecules which act as ligands for activating receptors. Thus, the lack of inhibitory signaling coupled with induction of activating signaling shifts the balance toward NK cell activation, leading to secretion of cytokines and killing of tumor cells.

Natural killer cell stimulation and effector function depend upon the integration of signals derived from two distinct types of receptors—activating and inhibitory receptors (Table 1). Normal healthy cells express MHC class I molecules on their surface which act as ligands for inhibitory receptors and contribute to the self-tolerance of NK cells. However, virus-infected cells or tumor cells lose surface MHC class I expression, leading to lower inhibitory signal in NK cells. Simultaneously, cellular stress associated with viral infection or tumor development such as DNA damage response, senescence program or tumor suppressor genes upregulate ligands for activating receptors in these cells. As a result, the signal from activating receptors in NK cell shifts the balance toward NK cell activation and elimination of target cells directly through NK cell-mediated cytotoxicity or indirectly through secretion of pro-inflammatory cytokines (10) (Figure 1).

**Table 1:** Activating and inhibitory receptors on NK cells.

Type	Receptors	Ligands	Species
Activating receptors	NKG2D	Mouse: Rae1a-e, MULT-1, H60	Mouse/human
		Human: MIC-A/-B, ULBP1–4	
	CD94-NKG2C	Mouse: Qa1b	Mouse/human
		Human: HLA-E	
	Ly49D	Mouse: H-2D^d^	Mouse
	Ly49H	Mouse: m157 of MCMV	Mouse
	KIR2DL4	Human: HLA-G	Human
	KIR2DS1	Human: HLA-C2	Human
	KIR2DS2	Human: HLA-C1	Human
	KIR2DS3	Unknown	Human
	KIR2DS4	Human: HLA-A11	Human
	KIR2DS5	Unknown	Human
	KIR3DS1	Human: HLA-Bw4	Human
	NKp30	Human: B7H6, BAT3, pp65 of HCMV, PfEMP1 of *Plasmodium falciparum*, viral HA	Human
	NKp46	Heparin, viral HA and HN	Mouse/human
	NKp44	Viral HA and HN, PCNA, proteoglycans	Human
	NKR-P1C		Mouse
	NKR-P1F	Mouse: Clr-g, Clr-c	Mouse
	NKR-P1G	Mouse: Clr-g, Clr-f	Mouse
	DNAM-1	Mouse and human: CD112, CD155	Mouse/human

Inhibitory receptors	Ly49A	Mouse: H-2D^b,d,k,p^, H-2M3	Mouse
	Ly49C	Mouse: H-2D^b,d,k^ H-2K^b,d,k^ m157	Mouse
	Ly49I	Mouse: H-2k^b,s,q,v^	Mouse
	Ly49P	Mouse: H-2D^d,k^	Mouse
	KIR2DL1	Human: HLA-C2	Human
	KIR2DL2	Human: HLA-C1	Human
	KIR2DL3	Human: HLA-C1	Human
	KIR3DL1	Human: HLA-Bw4	Human
	KIR3DL2	Human: HLA-A3,-A11	Human
	NKR-P1A	Human: LLTI	Human
	NKR-P1B	Mouse: Clr-b	Mouse
	NKR-P1D		
	CD94-NKG2A	Mouse: Qa1b	Mouse/human
		Human: HLA-E	
	ILT2 (CD85j)	Human: HLA-A, -B, -C, HLA-G1, HCMV UL18	Human
	CD244(2B4)	Mouse and human: CD48	Mouse/human

### Inhibitory Receptors on NK Cells

**Figure 2:**
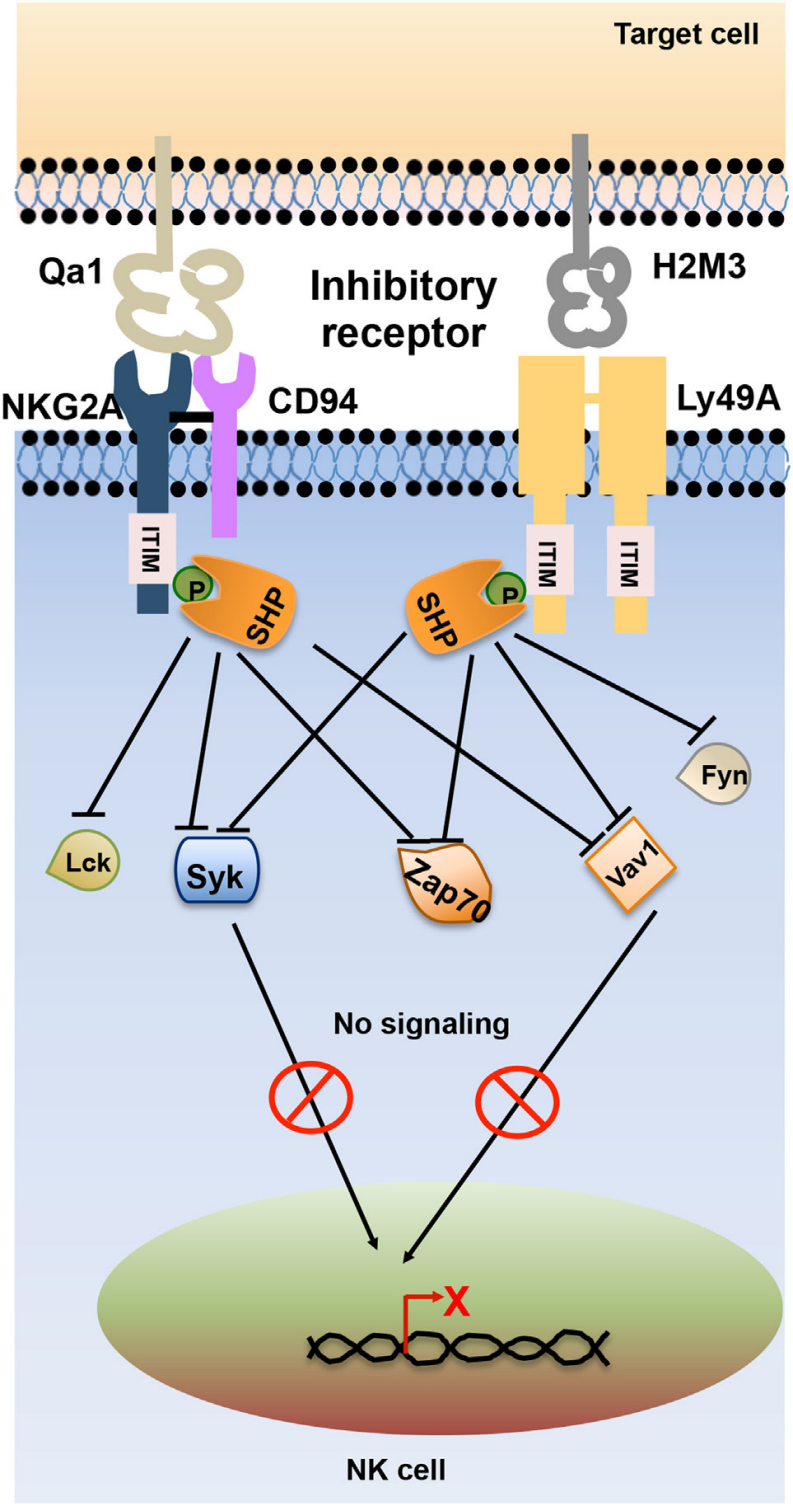
Schematic representation of natural killer (NK) cell inhibitory receptor signaling. The interaction of NK cell inhibitory receptors natural-killer group 2, member A (NKG2A) and Ly49A with its cognate ligand leads to phosphorylation of immunoreceptor tyrosine-based inhibitory motif (ITIM) in their cytoplasmic tails. Phosphorylated ITIM recruits phosphatases such as Src homology domain-containing tyrosine phosphatase (SHP) and SH2 domain-containing inositol-5-phosphatase (SHIP) that dephosphorylate signaling molecules such as Lck, Fyn, Syk, Zap70, and Vav1, thereby terminating activating receptor signaling in NK cells.

Inhibitory receptors signal through immunoreceptor tyrosine-based inhibitory motifs (ITIM) present in their cytoplasmic tails. Upon ligand engagement, ITIMs undergo phosphorylation and recruit phosphatases such as Src homology-containing tyrosine phosphatase 1 (SHP-1), SHP-2, and lipid phosphatase SH2 domain-containing inositol-5-phosphatase (SHIP) which further neutralize the activating signals (11). During NK cell inhibitory signaling, the phosphatases SHP-1 and SHP-2 dephosphorylate the immunoreceptor tyrosine-based activation motif (ITAM)-bearing Vav-1 molecules and prevent the downstream signaling (12, 13) (Figure 2).

Ly49 receptors represent one of the major families of mouse NK cell inhibitory receptors. Ly49 receptors are type II glycoprotein of C-type lectin-like superfamily and composed of carboxy-terminal lectin domain also known as NK domain (NKD) (14). Ly49 receptors bind to MHC class I molecules through their NKD, and this interaction is MHC-peptide independent. The Ly49 family of proteins are highly polymorphic which results in heterogeneous expression among different inbred mouse strains. The Ly49 receptor family includes Ly49A, Ly49C, Ly49I, and Ly49P molecules. The prototype member Ly49A binds to H-2D^d^, H-2D^k^, and the non-classical MHC-I molecule H2-M3 while Ly49C binds to H-2K^b^ and H-2D^b^ molecules (15). Human express functionally equivalent homolog of Ly49 member which are known as killer cell immunoglobulin-like receptor (KIR) family of proteins (16). KIRs are type I transmembrane protein with two or three IgG-like domains and a short or long cytoplasmic tail. KIRs bind to HLA-A, -B, and -C molecules. In contrast to Ly49 family, KIRs bind to the peptide-binding region of HLA molecules. The heterogeneity of KIR repertoire expression among different individuals is due to the difference in the expression of KIR molecules on individual NK cells as well as allelic variation in KIR genes. Inhibitory KIRs include KIR2DL1–3, KIR2DL5, and KIR3DL1–3 (17). Both KIRs and inhibitory Ly49 receptors contribute to NK cell tolerance to self-tissues (18). CD94-natural-killer group 2, member A (NKG2A) is another C-type lectin family of the inhibitory receptor that expresses as a heterodimer and contain ITIM. This receptor specifically recognizes non-classical MHC molecules on target cells and protect host cell against inappropriate NK cell activation (19, 20). Human NKG2A recognizes non-classical MHC molecule HLA-E (21, 22) while mouse counterpart interacts with the Qa1 molecule (23). There are several cytokines present in the tissue microenvironment that can modulate the expression of NKG2A and affect NK cell function.

### Activating Receptors on NK Cells

**Figure 3:**
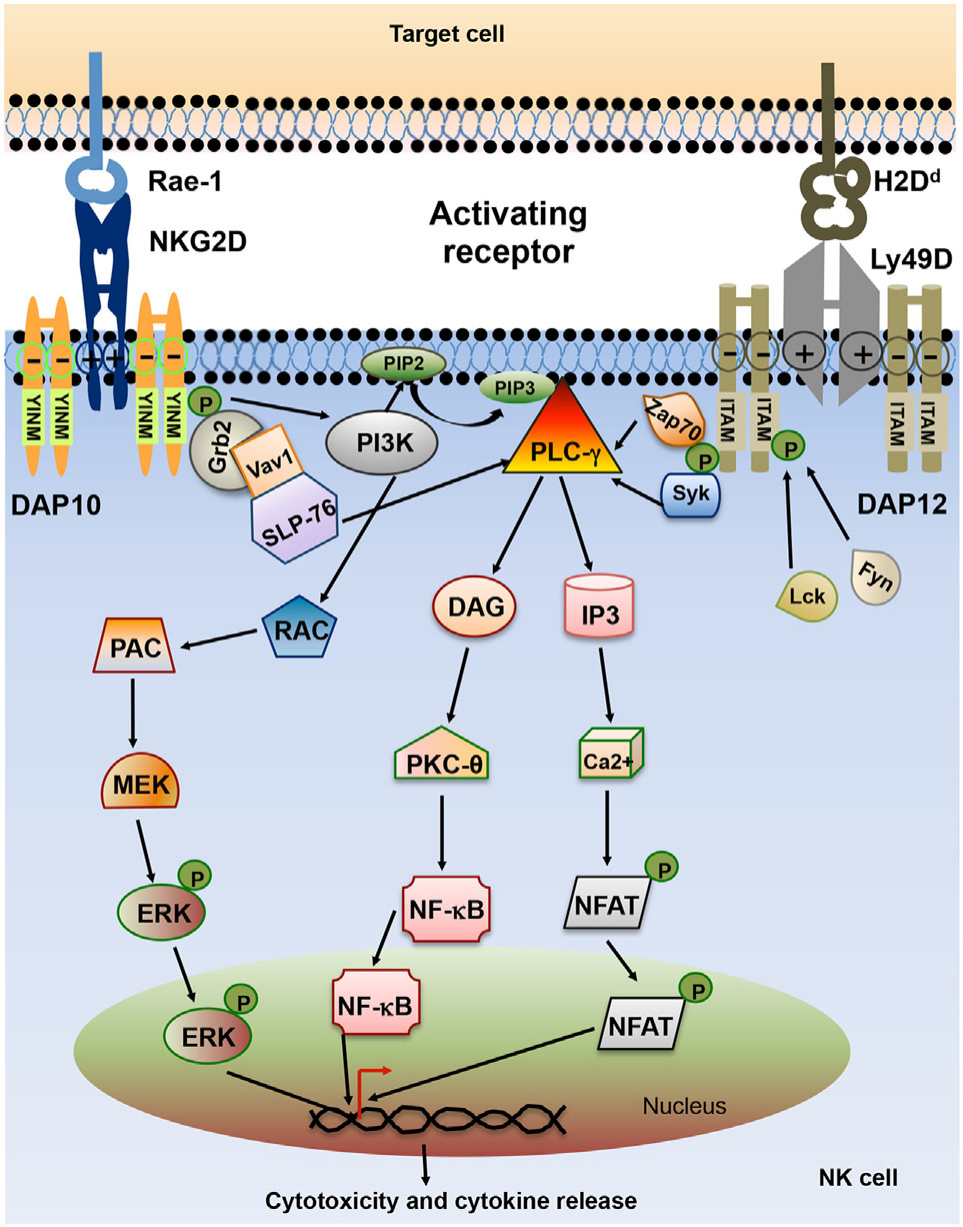
Schematic representation of natural killer (NK) cell activating receptor signaling. Interaction of activating receptor NKG2D and Ly49D with their cognate ligand leads to phosphorylation of YINM motif or immunoreceptor tyrosine-based activation motifs (ITAMs) present in the cytoplasmic tails of associated adapter protein such as DAP10 and DAP12. Phosphorylated ITAM or YINM motif recruits Syk/Zap70, PI3K, and Grb2/Vav1/SLP-76 complex. Grb2/Vav1/SLP-76 pathway activation leads to downstream activation of MEK/extracellular signal-regulated kinase (ERK) pathway. Phosphorylated Syk recruits PLC-γ which in turn activates inducible protein-3 (IP-3) and DAG pathway leading to activation of transcription factors NF-κB and NFAT. The net result of this signaling is the release of cytokines and chemokines as well as cytotoxic molecules by the NK cells (24).

Lack of MHC class I on the target cell is not sufficient to trigger NK cell activation. Full NK cell activation also requires recognition of stress-induced molecules by NK cell activating receptors. The effector function of NK cell utilizes integrated signaling from an array of activating receptors on NK cells (Table 1). Most activating receptors signal through ITAMs defined by the sequence D/EXXYXX(L/I)X_6–8_YXXL/I (where X_6–8_ is 6–8 amino acids stretch between two XXL/I element). Engagement of receptor-ligand complexes leads to phosphorylation of ITAM by Src family of tyrosine kinases such as Lck, Fyn, Src, Yes, Fgr, and Lyn. Phosphorylation of ITAM subunit leads to recruitment and activation of the tyrosine kinase Syk and Zap70. The downstream signaling pathway of Zap70 phosphorylation involves phosphorylation of different proteins such as SLP-76, Shc, and phosphatidylinositol-3-OH kinase [PI(3)K], assembly of Grb2, linker for the activation of T cells (LAT), Vav-1, and Vav-2, activation of mitogen-activated protein kinases (MAPKs) and extracellular signal-regulated kinases (ERKs). The outcome of these signals result in the elevation of calcium levels and reorganization of actin cytoskeleton leading to release of cytolytic granules containing perforin and granzymes and transcription of cytokine and chemokine genes (24) (Figure 3).

The activating receptor natural-killer group 2, member D (NKG2D) is a C-type lectin-like type II transmembrane protein expressed as a homodimer on the surface of all murine and human NK cells. It is also expressed by most NKT cells and activated CD8^+^ T cells in mice, and all CD8^+^ T cells and a subset of γδ T cells in humans (25). The NKG2D receptor is a hexameric complex composed of single NKG2D homodimer along with two DAP10 homodimers (26). In mice, NKG2D is present in two different isoforms, short isoform (NKG2D-S) and a long isoform (NKG2D-L), which are generated by alternative splicing (27). NKG2D-S can associate with both DAP10 and DAP12 while NKG2D-L only pair with DAP10. NKG2D-S is not present in human and NKG2D-L can only associate with DAP10 in human (27). DAP10 cytoplasmic domain contains YINM motif which is phosphorylated by Src family of kinases or Jak3 kinase and recruits p85 subunit of PI3K or Grb2 adaptor protein. Grb2 phosphorylation induces phosphorylation of Vav1, PLC-γ2, and SLP-76. PI3K and Grb-Vav1 signaling induce phosphorylation of Jak2, STAT5, Akt, MEK1/2, and Erk (28–31). Effector function of DAP10 signaling in NK cell is distinct from DAP12 signaling. Deficiency of DAP10 and DAP12 in mice and human NK cells has shown that NKG2D-DAP12 signaling can induce both cytotoxicity and cytokine secretion whereas signaling through DAP10 mostly activates cytotoxicity (27, 32, 33). The cytokines such as IL-7, IL-12, and IL-15 upregulate the expression of NKG2D on NK and CD8^+^ T cells, whereas IFN-γ and transforming growth factor-β (TGF-β) reduce it (34–36). The NKG2D molecule in human and mice binds several ligands which are highly polymorphic and are structural homologs of MHC class I molecules. The ligands of murine NKG2D are retinoic acid early inducible-1 family of proteins (Rae-1α-ε), murine UL16-binding protein-like transcript 1 (MULT1) and H60 group of proteins (H60a, H60b, H60c) (37–39). There are two families of NKG2D ligands in human, MHC class I chain-related protein A (MICA) and B (MICB) and UL16-binding proteins (ULBP1-6) (40). NKG2D ligands are expressed at low levels in healthy adult cells in both mice and human (40, 41). However, these ligands are upregulated and widely expressed in tumors of diverse tissue origin. Mouse NKG2D ligands have been detected in lymphoma cells, lung, colon, rectal and prostate cancer cell lines (41–43). However, lymphoma cell lines RMA and RMA-S, and melanoma cell line B16F10 lack expression of NKG2D ligands (37). A wide range of tumors such as leukemia, glioma, neuroblastoma (NB), melanoma, breast, lung, colon, kidney, and prostate tumors are known to express human MIC and ULBP (44–48). The level of expression of NKG2D ligands varies significantly between tumor types and stages of tumor progression. Ataxia telangiectasia mutated kinase and Rad3-related kinase (ATR), DNA damage repair pathway, cytokines, and TLR signaling are known to regulate the expression of NKG2D ligands (49–51). Pharmacological drugs such as proteasome inhibitors and histone deacetylase inhibitors also control NKG2D ligand expression (52, 53). In addition to NKG2D, two other members of NKG2 family, NKG2C and NKG2E act as an activating receptor and are known to express as a heterodimer with the CD94 molecule (19, 54). CD94-NKG2C and CD94-NKG2E heterodimer recognize class Ib molecule Qa-1^b^ and interact with DAP12 and activate downstream signaling (55, 56).

Although the majority of Ly49 receptors are inhibitory in nature, some Ly49 receptors such as Ly49D and Ly49H show activation function in C57BL/6 mice. Ly49D and Ly49H associated with the DAP12 molecule and transduce the signal through ITAM (57). Activation of the Ly49 receptor leads to phosphorylation of ITAM and recruitment of Syk tyrosine kinase (58). Ly49 receptors on NK cells play a critical role in host defense against viral infection. Ly49H binds to the glycoprotein m157 protein of murine cytomegalovirus (MCMV) and imparts resistance to MCMV infection (59). Other Ly49 activating receptors such as Ly49D, Ly49P, and Ly49W are reported to bind H-2D^d^ molecules (60–62). The activating KIR utilizes DAP12 adapter molecule for downstream signaling. The generation of activating Ly49 and KIR molecules was thought to be a result of convergent evolution from their respective ancestral inhibitory receptors (63).

Natural cytotoxicity receptors (NCRs) are another immunoglobulin superfamily of activating receptors that utilize extracellular immunoglobulin-like domain for ligand binding. Human NK cells express three distinct types of NCRs such as NKp46 (NCR1 or CD335), NKp44 (NCR2 or CD336), and NKp30 (NCR3 or CD337) while mouse NK cells express only NKp46 (64–66). NKp46 and NKp30 are expressed on both resting and activated NK cells, whereas NKp44 expression is restricted to activated NK cells. NCRs can bind to adaptor proteins FcεRI-γ and CD3-ζ which then transduce the signal through ITAM (67). NCRs recognize a wide variety of ligands on target cells ranging from viral, bacterial and parasite proteins to molecules from tumor cells and other host cells (67).

The 2B4 receptor (CD244) present in mouse and human NK cells belong to signaling lymphocyte activation molecule family of membrane receptors and depending on the adapter protein recruited at the cytoplasmic tail they can act as activating as well as an inhibitory receptor. CD244 predominantly acts as an inhibitory receptor in mice. NK cells from CD244 deficient mice were shown to have enhanced cytotoxicity and cytokine secretion and also help in efficiently rejecting the B16F10 melanoma (68, 69). However, neutralizing antibodies to human CD244 blocks the killing of CD48-expressing target cells. It has been shown that mouse cell lines transfected with human CD48 can be efficiently targeted and killed by human NK cells. These studies suggest that CD244 acts as an activating receptor in human NK cells (70, 71). The activating or inhibitory function of CD244 in human and mice may be influenced by other inhibitory and activating receptor signals and can also be perturbed by the relative expression of SAP, EAT-2, and ERT molecules involved in the downstream signaling.

CD38 is an enzyme that catalyzes the conversion of beta-Necotinamide adenine dinucleotide (beta-NAD^+^) and beta-necotinamide adenine dinucleotide 2’-phosphate (beta-NADP^+^)into cyclic adenosine diphosphate-ribose (ADPR) and nicotinic acid adenine dinucleotide phosphate (NAADP). CD38 has been shown to trigger the cytotoxic activity of NK cells against tumor cells by promoting the granule polarization and degranulation in NK cells. The ADPR produced by CD38 gets localized to cytolytic granules in response to stimulation and modulates Ca^++^ signaling, thereby causing degranulation in NK cells (72). Another study by Mallone et al. showed that CD38 engagement by agonistic monoclonal antibody (mAb) induces phosphorylation of CD3-ζ, FcεRI and ZAP-70 proteins leading to release of IFN-γ and GM-CSF and lysis of target cells (73). CD44 is also constitutively expressed by resting NK cells. However, stimulation of NK cells with IL-2 or IL-15 leads to upregulation and activation of CD44. The low molecular weight hyaluronic acid in combination with IL-2, IL-12, or IL-18 could trigger activated CD44 and promote IFN-γ production in NK cells (74). Crosslinking of CD44 with the mAb on NK cells also induces TNF-α production and CD16-mediated NK cell cytotoxicity (75).

The triggering of cytokines and chemokines secretion by NK cell and NK cell-mediated cytotoxicity requires a synergistic combination of several receptors. Using cross-linking antibodies to NK receptors, it has been shown that only CD16 alone could trigger degranulation of resting human NK cells while most activating receptors such as NKG2D and NCRs could perform activation only in combination with other receptors (76, 77). This synergistic activation of several receptors leads to convergence of signals toward a central signaling molecule so that its level reaches the threshold required for activation of NK cells.

### Costimulatory Receptors on NK Cells

Costimulatory receptors synergize with other activating receptors to provide additional stimulation. NKR-P1 in mouse acts as activating as well as an inhibitory costimulatory receptor. NKR-P1 is a member of type II glycoprotein receptors of C-type lectin superfamily and consist of five members: NKR-P1A, -B, -C, -D, and -E. NKR-P1C is mostly known to be associated with NK1.1 molecules and provides activating signal whereas NKR-P1B and -D contain ITIM and display inhibitory function (78, 79). Another costimulatory activating receptor is DNAX accessory molecule-1 (DNAM-1) or CD226, a member of Ig superfamily. DNAM-1 recognizes CD155 (also known as Poliovirus receptor or PVR) and CD112 (Nectin-2) on tumor cells and induces NK cell-mediated lysis (80). DNAM-1 has been shown to bind lymphocyte function-associated antigen 1 and promote adhesion of monocytes suggesting that DNAM-1 also plays a significant role in NK cell migration (81).

## Cytotoxic and Effector Immune Response of NK Cells

### Cytotoxic Immune Response of NK Cells

**Figure 4:**
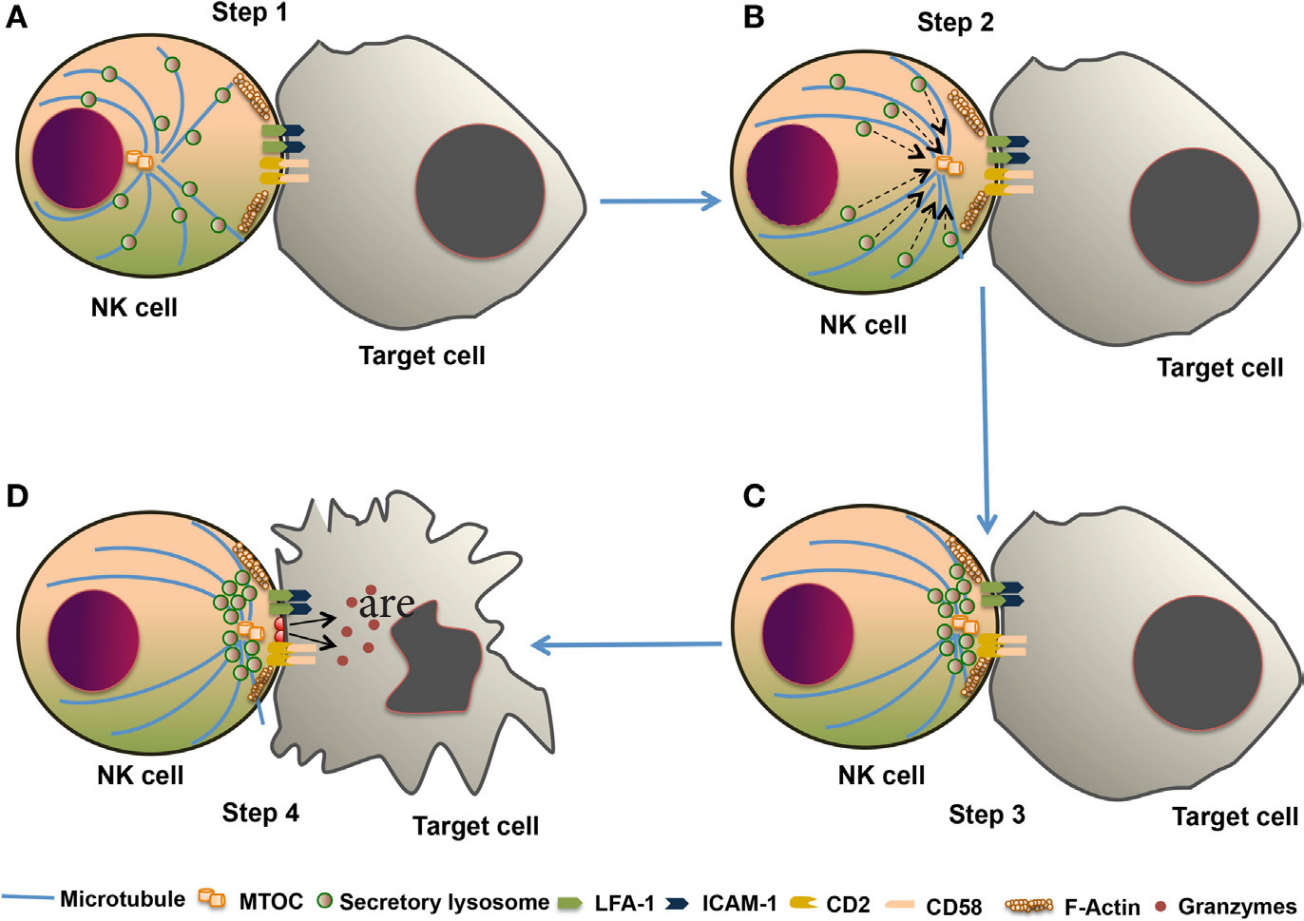
The cytotoxic response of natural killer (NK) cells. The NK cell cytotoxic response is tightly regulated in four discrete stages. **(A)** Step 1: Recognition of target cells by NK cell results in the reorganization of actin cytoskeleton and the formation of immunological synapse, and clustering of cell adhesion molecules such as lymphocyte function-associated antigen 1 (LFA-1) and CD2. **(B)** Step 2: microtubule organizing center (MTOC) and secretory lysosome polarize toward the immunological synapse. **(C)** Step 3: docking which involves moves close to the plasma membrane of NK cell at the synapse. **(D)** Step 4: secretory lysosome fuse with the target cell plasma membrane and releases the cytotoxic granules into the target cell.

The NK cell cytotoxic response is divided into four major steps. (1) Formation of immunological synapse between the target cell and NK cell, followed by reorganization of actin cytoskeleton. (2) Polarization of microtubule organizing center (MTOC) and secretory lysosome toward lytic synapse. (3) Docking of secretory lysosome with the plasma membrane of NK cells. (4) Fusion of secretory lysosome with the plasma membrane of target cells. This entire process leading to the release of cytotoxic molecules such as perforin and granzyme is known as degranulation. This degranulation of NK cells is often used for indirect measurement of NK cell cytotoxic activity (82) (Figure 4). During the NK cell degranulation, lysosomal-associated membrane protein-1 (LAMP-1 or CD107a) and -2 (LAMP-2 or CD107b) transiently appears on the surface of NK cells. The expression of LAMP-1 on NK cell surface has been used as an indirect measurement of NK cells cytolytic function (83). Perforin released in the target cells polymerizes and forms the pores, and facilitating the entry of granzymes into the target cell. Granzymes are serine proteases which activate caspase molecules leading to induction of apoptosis of target cells (24, 82, 84). Perforin-dependent cytotoxicity is crucial for NK cell-mediated control of several tumors (85, 86).

Another process by which NK cell mediates killing of target cells involves death receptor-induced target cell apoptosis. NK cells express TNF receptor ligand—Fas ligand (FasL), TNF, and TRAIL which binds to their corresponding receptor on target cells (87). Engagement of death receptor with its cognate ligand induces a conformational change in the receptor and recruitment of adaptor protein leading to apoptosis of target cells (88, 89). NK cell-mediated control of methylcholanthrene (MCA) metastasis has been shown to be TRAIL dependent (90). Fas-FasL pathway contributes to the antimetastatic potential of IL-18-treated NK cells (91). These studies suggest that NK cells use various molecules to induce the cytotoxic function against physiologically stressed cells.

It has been shown that exosomes produced by immune cells can promote the antitumor immunity whereas tumor cell-derived exosomes in tumor microenvironment can inhibit the effector immune response (92). Recent studies have shown that both resting and activated human NK cells secrete exosomes that have NK cell-specific marker CD56 and several other NK cell-associated molecules such as NKp30, NKp44, NKG2D, and NKp46. These exosomes also have FasL and perforin molecules and exert cytotoxic activity against various human tumor cell lines (93). Another study reported that exosomes produced by NK cells that have been pre-exposed to NB cells (Nx-ANKs) show higher expression of different activating receptors such as NKp30, NKp44, NKp46, and NKG2D and also have enhanced cytotoxicity when compared with untreated NK cells (92). These findings suggest that NK cells pre-exposed to NB cell-derived exosomes have undergone education which results in efficient cytotoxicity against NB tumors (92).

### Effector Immune Response of NK Cells

Activated NK cells secrete a wide variety of cytokines such as IFN-γ, TNF-α, GM-CSF, IL-10, IL-5, and IL-13 and chemokines such as MIP-1α, MIP-1β, IL-8, and RANTES (94–96). IFN-γ is one of the most potent effector cytokines secreted by NK cells and plays a crucial role in antiviral, antibacterial, and antitumor activity. IFN-γ has been shown to modulate caspase, FasL, and TRAIL expression and activates antitumor immunity (97). The tumor stromal cells control the secretion of effector cytokines in NK cells. Signaling through activating receptor NKG2D on NK cell has been shown to promote the release of IFN-γ (98). IL-12 produced by dendritic cells, macrophages, and neutrophils can also induce the production of IFN-γ in NK cells, which could be further enhanced by TNF-α, IL-1, and IL-18 (99, 100). In contrast, TGF-β inhibits the production of IFN-γ, TNF-α, and GM-CSF in NK cells (101). IL-10 is also a potent inducer of NK cell proliferation, cytotoxic function, and IFN-γ production in combination with IL-18 (102). Treatment with IL-18 promotes regression of melanoma tumor in the NK cell-dependent manner (103). IL-12 treatment inhibits tumor metastasis in the NKG2D and perforin-dependent manner, while the antimetastatic effect of IL-18 in the same setting is FasL dependent (43). IL-21 has also been known to induce NK cell activation *in vivo* in melanoma and renal cell carcinoma patients and also mediate rejection of various murine tumors in a NKG2D-dependent manner (104, 105). IL-15 is known to activate NK cell function and suppress tumor growth. These studies point out that apart from the NK cell cytotoxic function, cytokines secreted by the NK cells also provide a significant boost to the antitumor immunity. Similarly, the cytokines secreted by other immune cells or stromal cells in the tumor microenvironment can positively or negatively influence the antitumor function of NK cells.

## Tolerogenic and Inflammatory Function of NK Cells

### NK Cell Tolerance and Education

Natural killer cell tolerance to self-molecules is dependent on recognition of MHC class I molecules on target cells by inhibitory receptors present on NK cells. Many of the activating receptors expressed by mouse and human NK cells recognize self-ligands, thus raising the possibility of autoreactivity unless restrained by inhibitory receptors. When NK cells develop in the presence of self-ligand for the activating receptor, they are tolerant toward the specific activating receptor. The activating receptor Ly49D recognizes MHC class I molecule H-2D^d^. When NK cells develop in mice lacking H-2D^d^, they are able to kill H-2D^d^-expressing target cells. However, Ly49D^+^ NK cells from H-2D^d^-expressing mice show tolerance toward H-2D^d^-expressing target cells (106). One possible mechanism for this self-tolerance is the coexpression of H-2D^d^ recognizing inhibitory receptors Ly49A and Ly49G2 along with Ly49D on NK cells. The Rae-1 family of ligands that bind to the activating receptor NKG2D are known to be constitutively expressed in the embryos but absent in healthy adult tissues. Adoptive transfer of bone marrow cells from Rae-1ε transgenic mice to syngeneic wild-type mice leads to efficient rejection of adoptively transferred NK cells (107). However, NK cells from Rae-1ε transgenic mice do not kill Rae-1-expressing tumor cells suggesting that NK cells developed in the presence of ligands for the specific activating receptor NKG2D show tolerogenic phenotype toward cells expressing those ligands (108).

The importance of inhibitory receptor-MHC class I engagement in NK cell tolerance and education can be understood from the fact that NK cells which develop in the absence of MHC class I molecules do not kill MHC class I-deficient tumor cell lines or reject MHC class I-deficient allogeneic bone marrow cells *in vivo* (109, 110). The types of inhibitory receptor expression on NK cells are varied and stochastic such that various populations of NK cell have a distinct combination of inhibitory receptors. Recent studies suggested that a significant number of NK cells in mouse and human either lack expression of any self-MHC-specific inhibitory receptors or express receptors specific for non-self-MHC class I. These subsets of NK cell are non-responsive to several activating receptor stimulations *in vitro* and fail to reject MHC class I-deficient bone marrow cells *in vivo* (111, 112). Thus, engagement of self-MHC class I with inhibitory receptor during NK cell development is necessary for full responsiveness of activating receptors and rejection of MHC-deficient cells and this process is known as NK cell education.

Several mechanisms have been proposed to explain NK cell education, one of the models being disarming model. According to this model, NK cells are by default responsive and become tolerant to normal cells after the acquisition of self-MHC-specific inhibitory receptor. The presence of activation pathways allows NK cells to reject target cells that lose MHC I molecules or upregulate ligands for activating receptors. However, if the NK cell fails to acquire self-MHC class I-specific inhibitory receptor, chronic stimulation by normal cells makes them hyporesponsive (113). In support of this model, it has been observed that transgenic C57BL/6 mice expressing H-2D^d^ are able to reject C57BL/6 bone marrow cells (express H2-D^b^) while transgenic H-2D^d^ mice having a mosaic expression of H-2D^d^ and H-2D^b^ are unable to reject C57BL/6 bone marrow cells (114). The other model, known as licensing or arming model suggests that NK cells are initially hyporesponsive and become licensed or armed into effector cells after engagement of their inhibitory receptors with MHC class I during development. The fact that NK cell education does not require SHP-1 and SHIP-1 phosphatases suggests that inhibitory signals are indispensable for NK cell education and supports arming model (115). In addition to these, another model known as tuning or rheostat model is proposed where NK cell response is tuned by the number of self-MHC class I-specific receptor expressed on NK cell and the affinity of its cognate receptors. Thus, according to this model, NK cell education is a quantitative process, which depends on the strength of activating or inhibitory signal received by NK cell. If the strength of inhibitory signaling opposes chronic activating receptor stimuli, then NK cells are maintained in the highest responsive state. In contrast, strong stimulation without opposing inhibitory signal sends NK cells to lowest responsive state while intermediate net stimulation supports medium responsiveness (116).

### NK Cell Memory and Antitumor Immunity

Although NK cell is traditionally considered as a part of the innate immune system, now it has been shown that these cells also display memory cell-like features (117). NK cell memory response has been reported in three circumstances—antigen-specific memory NK cells in the liver, CMV-specific NK cells and cytokine-induced memory-like NK cells (118). Liver resident memory NK cells mediate hapten-specific contact hypersensitivity response and this reaction is abrogated in mice deficient for IL-12, IFN-γ, or IFN-αR signaling (119). Liver memory NK cell response to the melanocyte-specific prohapten monobenzone is dependent on macrophage activation through inflammasome NLRP3 and IL-18 (119). The memory NK cell response is also reported in MCMV virus infection. Ly49H^+^ NK cells recognize the m157 protein of MCMV virus and are capable of forming long-lasting memory following MCMV reinfection (120). In human, memory NK cells respond to human CMV (HCMV) virus is known to express a high level of activating receptor NKG2C, and these NKG2C^+^ NK cells expand during acute infection as well as during secondary challenge (121). Murine cytokine-induced memory NK cells initially activated with a high dose of IL-12 and IL-18 were shown to have increased cytokine secretion when restimulated after two weeks of primary antigen challenge (122). Similarly, human cytokine-induced memory NK cells with long-term effector response have been reported in response to IL-12, IL-15, and IL-18 stimulation (123).

Antitumor effect of memory NK cell has been studied mostly in HCMV model. CD56^dim^NKG2C^+^ NK cells from a HCMV^+^ donor are shown to have increased TNF-α and IFN-γ production in response to K562 tumor cell stimulation. These cells preferentially expand during HCMV reactivation in hematopoietic cell transplantation (HCT) recipients and play a significant role in mediating relapse protection with the better post-HCT outcome (124). Adoptively transferred IL-12-, IL-15- or IL-18-activated murine NK cells are shown to display memory features and inhibit tumor growth in IFN-γ and perforin-dependent manner. These preactivated NK cells possess demethylation of CpG residue in the CNS1 region of IFN-γ locus and show antitumor activity (125, 126). Human IL-12-, IL-15-, and IL-18-induced memory-like NK cells show higher expression of granzyme B and perforin and display enhanced cytotoxicity against K562 tumor cells (123). These NK cells are also shown to have increased TNF-α and IFN-γ production in response to primary acute myeloid leukemia (AML) blasts and control AML growth in mice. Furthermore, its therapeutic use resulted in complete remission of nine AML patients in phase I clinical trial (127). Thus, harnessing the potential of NK cell memory for therapeutic purpose remains a promising translational approach to control tumor growth in the clinic.

## NK Cell-Based Cancer Immunotherapy

### NK Cells in Cancer Immunosurveillance

Natural killer cells play a pivotal role in cancer immunosurveillance and also cooperate with other adoptive immune cells for antitumor immunity (98, 128). Removal of NK cells has been shown to increase the incidence of MCA-induced sarcoma suggesting that NK cells are involved in tumor cell elimination (129). A study by O’Sullivan et al. provided further evidence for the role of NK cell in immunosurveillance where the incidence of MCA-induced sarcoma was greater in RAG2^−/−^γc^−/−^ mice (lacking both adaptive immunity and NK cells) when compared with RAG2^−/−^ mice (lacking only adaptive immunity) (130). The mechanisms of NK cell elimination of MCA-induced sarcomas involve molecules like NKG2D, IFN-γ, and perforin (97, 131). The perforin-dependent NK cell activity was reported to control B cell lymphomas and mammary carcinoma (132). In a mouse model of liver carcinoma, it was observed that restoration of endogenous p53 in tumor cells promote NK cell-mediated elimination of senescent tumor cells (133). However, many tumors escape NK cell attack and grow progressively. Tumor cells secrete immunosuppressive factors such as TGF-β, VEGF, indoleamine 2,3-dioxygenase (IDO), prostaglandin E2 (PGE2), and adenosine which inhibit antitumor immune functions (134). Pietra et al. have demonstrated that melanoma cell-derived IDO and PGE2 inhibit the cytolytic activity of NK cells *in vitro* (135). We have also shown that melanoma tumor-infiltrating NK cells downregulate several activating receptors, upregulate inhibitory receptors and display poor degranulation when compared with NK cells in the secondary lymphoid tissues (98). The intratumoral NK cells are also known to have reduced proinflammatory cytokines and cytokine receptors expression which might hamper their antitumor response in the tumor microenvironment (98). Melanoma-associated fibroblasts have been reported to suppress the cytotoxic activity of NK cells in both contact-dependent and contact-independent manner (136). Several other suppressive cell types such as regulatory T cells (Tregs) and myeloid-derived suppressor cells (MDSCs) can impair antitumor immune response by inhibiting the function of tumor-specific effector T cells. Tregs, MDSC, and M2-macrophages also known to inhibit the cytolytic function of intratumoral NK cells through the production of IL-10 and TGF-β (137–139).

### Adoptive NK Cell Therapy

**Figure 5:**
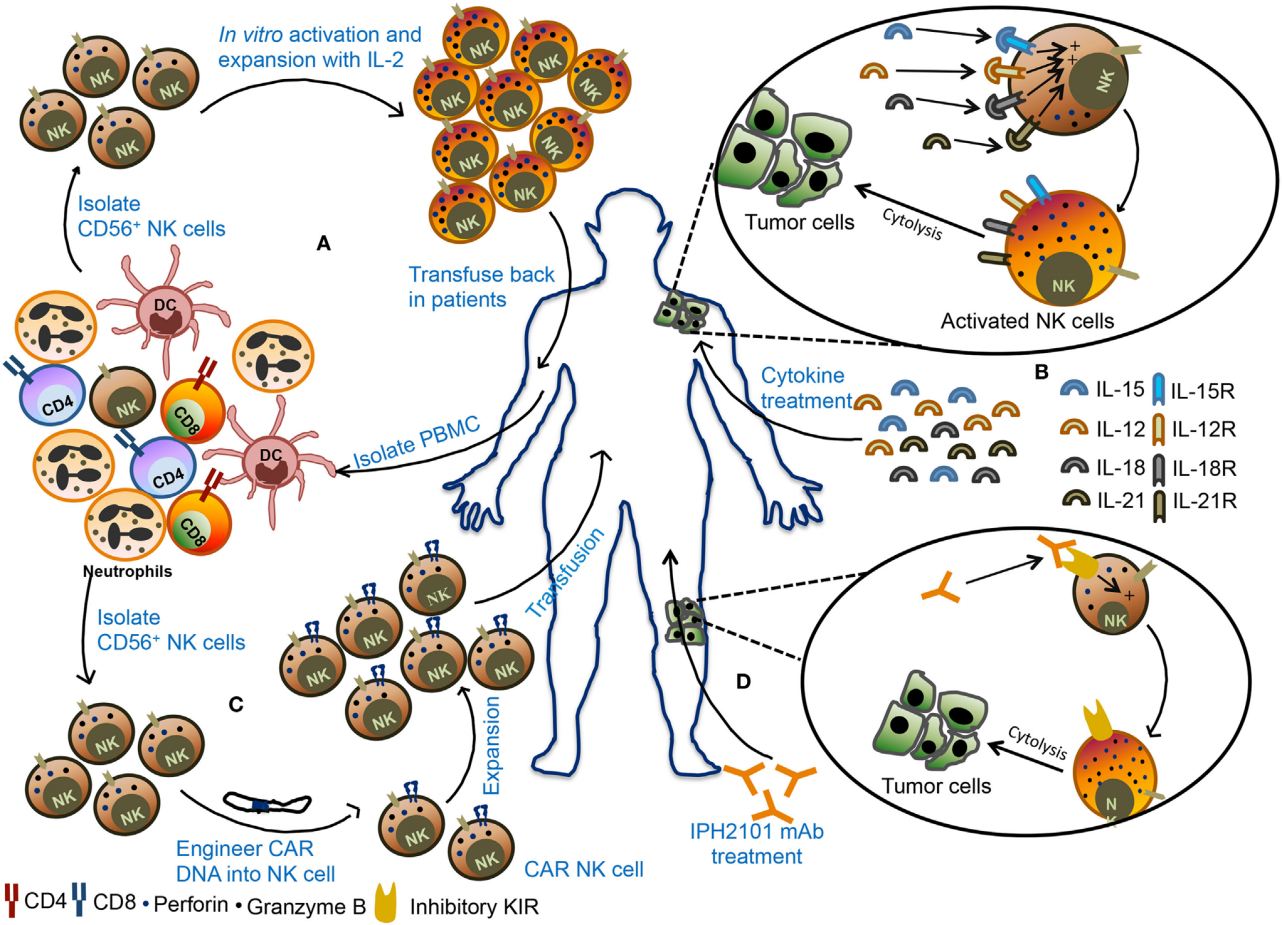
Various natural killer (NK) cell-based immunotherapy approaches. **(A)** In adoptive cellular therapy, NK cells are freshly isolated from peripheral blood mononuclear cells (PBMCs) from the patient. These NK cells are *ex vivo* activated and expanded and then transfused back into the patient. **(B)** In the second strategy, patients are treated with recombinant cytokines such as IL-2, IL-15, IL-12, or IL-18. These cytokines promote activation and proliferation of NK cells in the body leading to better antitumor immunity. **(C)** In the third strategy, NK cells are engineered to express chimeric antigen receptor (CAR), followed by expansion of these CAR NK cells, and transfused back into the patient. **(D)** In monoclonal antibody (mAb)-based treatment, patients are treated with IPH2101, a blocking mAb against inhibitory receptors and these mAb can promote NK cells activity in the tumor microenvironment and restrict tumor growth.

Harnessing NK cells for the therapeutic purpose is an attractive option and has received rejuvenating interest in recent times (Figure 5). NK cells immunotherapy offers several advantages. First, use of NK cells will bypass the need of antigen-specific T cells. Second, NK cells can directly kill tumor cells and can also rapidly secrete proinflammatory cytokines that can potentiate the adaptive immune response (128). Finally, NK cells are easy to isolate and manipulate and have a relatively short lifespan. Therefore, the possibility of overexpansion of transferred NK cells in the recipient’s body is less.

The source of NK cells for adoptive therapy can be autologous (from the same patient) or allogeneic (from other healthy donors). In autologous NK cell-based adoptive therapy, NK cells are isolated from patients using CD56 beads, activated *ex vivo* and transfused back into the same patient followed by administration of cytokines such as IL-2 to support their *in vivo* expansion and stimulation (Figure 5A). Transfusion of *ex vivo* activated and expanded autologous NK cells in breast cancer, lymphoma, renal cell carcinoma, metastatic melanoma, and gastrointestinal cancer patients did not lead to favorable anticancer response (140–142). In most cases, there was either an increase in circulating NK cells or *in vitro* cytokine release or cytotoxicity by adoptively transferred NK cells but it was not enough to mediate tumor regression in the patients. The failure of autologous NK cell adoptive therapy to produce clinical outcome insisted on the use of allogeneic NK cells for immunotherapy. The advantage of allogeneic NK cells over autologous ones is due to its less likely to be inhibited by NK cell-mediated recognition of self-MHC molecules. Adoptive transfer of haploidentical NK cells from KIR-mismatched donor has been shown to be safe and also mediate complete remission in AML patients (143, 144). In most clinical trials of NK cell-based immunotherapy, peripheral blood (PB) was utilized as a source of NK cells. However, alternative sources of NK cells such as bone marrow, hESCs, and cord blood (CB) NK cells can also be explored for their therapeutic benefits as a cellular therapy for cancer. Successful expansion of CB NK cells using artificial antigen presenting cells has been reported and shown to have *in vivo* antitumor activity against multiple myeloma (145, 146). In contrast to hematological malignancies, NK cell-based immunotherapy was found to be less promising for solid tumor. Early phase clinical trial using the infusion of activated allogeneic NK cells in ovarian and breast cancer patients showed transient donor chimerism but did not show a significant expansion of transfused NK cells (147). NK cell-based immunotherapy of solid tumor posed several challenges such as reduced infiltration of NK cells into tumor microenvironment, lack of susceptibility of tumor cells to NK cell cytotoxicity and alteration of NK cell function by suppressive immune cells. Combining NK cell-based immunotherapy with approaches that can target immunosuppressed tumor microenvironment may provide benefit in the treatment of solid tumors.

### Cytokine Therapy

Several cytokines such as IL-2, IL-15, IL-12, IL-18, and IL-21 can activate and boost NK cell function (Figure 5B) (148–151). Among these, IL-15 stands out to be the most promising cytokine to be used as an activator of NK cells. Infusion of IL-15 into the metastatic malignant patients in a phase I clinical trial showed proliferation and expansion of NK cell along with other antitumor immune cells such as CD8^+^ T cells and γδ T cells. This study establishes a safe tolerance of IL-15 in patients and also achieved reduction of lung lesions in two patients (152). The superagonist IL-15-1L-15Rα-Sushi-Fc has been shown to promote NK cell-mediated antitumor activity against breast, lung, and colon carcinomas in preclinical studies and holds a promising approach in the clinical trial (153, 154).

### Chimeric Antigen Receptor (CAR) NK Cell Therapy

Adoptive transfer of NK cells that are engineered to express CAR against a specific tumor antigen such as ganglioside GD2, 2B4 (CD244) receptor, CD138, and CS1 have also been tested in several preclinical models (Figure 5C). In these models, CAR-transduced NK cells demonstrated efficient killing of tumor cells *in vivo* and *in vitro* (155–158). Interestingly, NKG2D-DAP10-CD3ζ-expressing NK cells are also shown to have enhanced cytotoxic activity and cytokine secretion potential *in vitro* and display enhanced antitumor activity to osteosarcoma (159). Large-scale isolation and expansion of NK cells from human PB and difficulties in the transfection of PB NK cells have hindered the production of human CAR NK cells. For these reasons, the human NK92 cell line consisting of activated NK cells have emerged as a popular choice for use in NK cell immunotherapy because of their relative ease in transfection. NK92 cells engineered to express CD19 CAR are shown to specifically target CD19 expressing leukemia cells (160, 161). Similarly, NK-92 cells transduced to express both wild-type and mutated EGFR CAR display cytolytic activity as well as IFN-γ production against glioblastoma cells (162). NK-92 cells expressing receptor tyrosine kinase ErbB2 (HER2)-specific CAR were also shown to regress glioblastoma (GBM) tumors in an orthotopic GBM xenograft model (163). Currently, umbilical CB-derived CAR-engineered NK cells (CD19-CD28-zeta-2A-iCasp9-IL-15 transduced) are in clinical trial for relapsed/refractory CD19^+^ B lymphoid malignancies (NCT03056339). The NK-92 cell line engineered to express anti-CD33 or anti-CD7 linked to TCR zeta, CD28 and 4-1BB signaling domains are undergoing clinical trials for CD33^+^ AML (NCT02944162) or CD7^+^ relapsed or refractory leukemia and lymphoma (NCT02742727), respectively. These studies suggest that CAR NK cells have a strong potential to control the tumor growth that shows resistance to conventional immunotherapy.

### mAb-Based Therapy

Natural killer cells express various activating, costimulatory, and inhibitory receptors that can be targeted to improve NK cell cytotoxicity (Table 1). One such receptor is CD16, which binds to Fc region of antibodies and promotes antibody-dependent cell-mediated cytotoxicity (ADCC) of tumor cells. The combination of Rituximab and IL-2 has been shown to induce ADCC and increase NK cell activity (164). The ADCC activity of NK cells through CD16 can be enhanced by using bispecific or trispecific antibodies which incorporate Fv region recognizing tumor cell antigen along with Fv region that binds CD16. In the multiple myeloma patients, the effector immune cells do not easily recognize malignant plasma cells. To make these malignant cells as effective target to NK cell, a recombinant bispecific protein (ULBP2-BB4) were developed where ULBP2 interact with NKG2D on NK cells and BB4 moiety binds to CD138 expressed on the plasma cells. This bispecific fusion protein showed enhanced NK cell-mediated elimination of primary malignant plasma cells in the allogeneic and autologous setting and CD138^+^ human multiple myeloma cell lines, U-266 and RPMI-8226 (165). Administration of IL-15Rα chain in exosome along with NKG2D or NKp30 ligand to restore NK cell function has also been proved to be beneficial and resulted in progression-free survival in metastatic melanoma and non-small cell lung cancer patients (166). Further, blockade of inhibitory receptor signaling and immune checkpoints might serve as an alternative strategy to boost NK cell function (Figure 5D). Although tumor cells downregulate HLA class I, many tumor cells retain MHC class I molecules and their interaction with inhibitory receptors expressed on NK cells might dampen NK cell function. In such scenario, blockade of HLA-inhibitory receptor interaction might prove to be beneficial. IPH2101, a human IgG mAb which blocks signaling mediated by inhibitory receptor KIR2DL-1, -2, -3 showed enhanced NK cell activation and complete remission in AML patient in the phase I clinical trial but failed to show substantial clinical benefits in multiple myeloma patients in phase II trial (167, 168). Blocking of NKG2A-HLA-E interactions was also shown to increase NK cell cytotoxic activity (169). IPH2201, a blocking mAb to NKG2A is currently in clinical trial for solid tumors (NCT02671435 and NCT02643550). In future, the combinatorial approach targeting a different aspect of NK cell function might prove beneficial for the treatment of cancer.

## Conclusion

The understanding of the cellular and molecular biology of NK cell has paved way for the design of NK cell-based immunotherapeutic strategies. However, the majority of the clinical trials attempting NK cell-based immunotherapy in solid tumor (including adoptive transfer of autologous, allogeneic NK cells, NK cell lines or CAR NK cells, cytokine-based therapy, anticancer inhibitors, or agonist of activating receptors) have only achieved low efficacy. One of the primary challenges for NK cell-based immunotherapy for solid tumor is migration and persistence of cytotoxic NK cells into the tumor microenvironment. The chemokines expressed in the tumor microenvironment and cognate expression of chemokine receptor on NK cells help in the efficient mobilization of NK cells in the tumor. CXCR3 has been shown to regulate the accumulation of CD27^high^ NK cells in subcutaneous lymphoma (170). The chemokines required for the migration of NK cells in various tumor tissues need to be identified. For the better efficacy of adoptive NK cell therapy *in vitro* activated, manipulated or genetically-modified NK cell has to have an expression of cognate chemokine receptors in order to efficiently mobilize into the tumor microenvironment. The use of an agonist of chemokine receptors that promote NK cell migration in the tumor microenvironment and combining cytokines or small molecule drugs that promote the cytolytic function and migration of NK cell in the tumor microenvironment should be explored as an alternative strategy. Besides, methodologies for the adequate large-scale manufacturing of large numbers of NK cells for its clinical use need to be developed.

Emerging evidence also suggests that tumor cells modulate the NK cell phenotype and function. To address this, in depth knowledge of the suppressive network that impairs NK cell effector function in the tumor microenvironment is needed. A comprehensive study of NK cell signaling pathways should also be carried out to identify novel targets that can be used to improve the NK cell antitumor response. Particular emphasis should be given on identification of unique pathways or checkpoints of NK cell activation that can be exploited to develop small molecules that stimulate NK cell function in the tumor microenvironment. The patient receiving radiation therapy or chemotherapy as part of condition regimen before NK cell transfusion and its effect on NK cell function need to be critically evaluated. Thus, understanding the cellular and molecular biology of NK cells will allow us to identify novel molecules that could be used as immunotherapy.

## Author Contributions

SP and GL conceived the idea and wrote the manuscript.

## Conflict of Interest Statement

The authors declare that the research was conducted in the absence of any commercial or financial relationships that could be construed as a potential conflict of interest.

## Abbreviations

ADCC, antibody-dependent cell-mediated cytotoxicity; AML, acute myeloid leukemia; CAR, chimeric antigen receptor; CB, cord blood; CLP, common lymphoid progenitor; DNAM-1, DNAX accessory molecule-1; ERK, extracellular signal-regulated kinase; FasL, Fas ligand; GM-CSF, granulocyte macrophage colony-stimulating factor; HCT, hematopoietic cell transplantation; IFN-γ, interferon-γ; ITAM, immunoreceptor tyrosine-based activation motif; ITIM, immunoreceptor tyrosine-based inhibitory motif; MCA, methylcholanthrene; MCMV, murine cytomegalovirus; MDSCs, myeloid-derived suppressor cells; NCRs, natural cytotoxicity receptors; NK, natural killer; NKG2A, natural-killer group 2, member A; NKG2D, natural-killer group 2, member D; KIR, killer cell immunoglobulin-like receptor; TGF-β, transforming growth factor-β; PB, peripheral blood; Rae-1, retinoic acid early inducible-1; SHIP-1, Src homology 2-containing inositol 5′-polyphosphatase-1, SHP, Src homology domain-containing tyrosine phosphatase; TNF-α, tumor necrosis factor-α; TRAIL, TNF-related apoptosis-inducing ligand; Tregs, regulatory T cells.
